# Guidelines for Enhanced Recovery After Trauma and Intensive Care (ERATIC): Enhanced Recovery After Surgery (ERAS) and International Association for Trauma Surgery and Intensive Care (IATSIC) Society Recommendations: Paper 2: Postoperative and Intensive Care Recommendations

**DOI:** 10.1002/wjs.70004

**Published:** 2025-07-22

**Authors:** Timothy C. Hardcastle, Christine Gaarder, Zsolt Balogh, Scott D'amours, Kimberly A. Davis, Amit Gupta, Shahin Mohseni, Paal A. Naess, Shanisa Naidoo, Tarek Razek, Simon Robertson, Hayaki Uchino, David Zonies, Jade Whing, Michael J. Scott

**Affiliations:** ^1^ Inkosi Albert Luthuli Central Hospital Durban South Africa; ^2^ Department of Surgical Sciences University of KwaZulu‐Natal Durban South Africa; ^3^ Oslo University Hospital Ulleval and University of Oslo Oslo Norway; ^4^ John Hunter Hospital and University of Newcastle Newcastle Australia; ^5^ Liverpool Hospital and University of Sydney Sydney Australia; ^6^ Division of General Surgery Trauma and Surgical Critical Care Yale School of Medicine New Haven Connecticut USA; ^7^ JPN Apex Trauma Centre All India Institute of Medical Sciences New Delhi India; ^8^ Department of Surgery New Al Jahra Hospital Al Jahra Kuwait; ^9^ School of Medical Sciences Orebro University Orebro Sweden; ^10^ Montreal University Hospital and McGill University Montreal Canada; ^11^ Canberra Hospital Canberra Australia; ^12^ Department of Surgery University of Washington Seattle Washington USA; ^13^ Norfolk and Norwich University Hospital Norwich UK; ^14^ Department of Anesthesiology and Perioperative Medicine University of Rochester Rochester New York USA; ^15^ Leonard Davis Institute for Health Economics University of Pennsylvania Philadelphia Pennsylvania USA; ^16^ University College London London UK

**Keywords:** enhanced recovery after surgery, ERAS, major trauma, perioperative care, polytrauma

## Abstract

**Background:**

Enhanced recovery after surgery (ERAS) protocols reduce length of stay, complications, and costs for elective surgical procedures. It remains challenging to implement ERAS concepts in the acute trauma patient due to deranged physiological reserve from the penetrating or blunt trauma producing altered physiology. However, systems of care improve access to early intervention and potentially reduce mortality. These consensus guidelines examine optimal prehospital, resuscitation‐room, intraoperative and postoperative treatment, systems of ethical management, and overall care for trauma patients in the postresuscitation phase of care. The guideline is presented in three parts, this being Part 2.

**Methods:**

Experts in aspects of management of trauma surgical patients and intensive care were invited to contribute by the International ERAS Society and IATSIC. PubMed, Cochrane, Embase, and MEDLINE database searches on English language publications were performed for ERAS elements using the patient, intervention, comparator outcome (PICO) consensus questions created by the expert group. Studies were selected with particular attention to randomized clinical trials, systematic reviews, meta‐analyses, and large cohort studies; reviewed and summarized recommendations were graded using the Grading of Recommendations, Assessment, Development and Evaluation (GRADE) system. These recommendations based on current best evidence, with extrapolation from elective patient studies, where appropriate, were followed by a modified two‐round Delphi method to validate final recommendations. Several ERAS components are already standard of care within national and society guidelines and are endorsed. The bulk of the text focuses on key areas pertaining specifically to trauma care of major trauma and polytrauma in the ICU‐requiring group.

**Results:**

Overall, 37 aspects of trauma care were considered, with multiple PICO questions and subpoints. Consensus was reached after two rounds of a modified Delphi process involving all authors, with minor adjustments to some phrasing required, but with 87% overall agreement on all statements (100% agreement on 31 of the main statement sets, prior to minor edits to address the points of difference for the rest, with 100% total agreement thereafter). None were rejected outright. The recommendations and level of evidence for each aspect of trauma care that may impact on improved recovery and reduced length of hospital stay are presented with grade of recommendation.

**Conclusions:**

This paper presents the results of the postoperative care and ICU aspects. The guidelines are based on current best evidence for an ERAS approach to patients who have had major injuries and polytrauma. These guidelines are not exhaustive but collate the best available evidence on important components of care for this patient population. As some of the evidence is extrapolated from elective surgery and nontrauma emergency surgery, some of the components need further evaluation in future studies.

## 1 Introduction

This article continues the ERATIC guidelines for trauma and intensive care dealing with the postoperative care aspects of the polytrauma or major trauma patient in the intensive care unit, the wards during recovery, and in preparation for rehabilitation.

The background and detailed methods of the literature search and Delphi consensus process were detailed in Paper 1 and system aspects along with ethical matters are addressed in Paper 3. (The papers are found as https://doi.org/10.1002/wjs.70002 and https://doi.org/10.1002/wjs.70003 in the *World Journal of Surgery*.)

## 2 Section E—Postoperative and Intensive Care (ICU)

13 sepsis screening in the postoperative period

**PICO: In trauma patients in the postoperative period, what are the ICU sepsis screening tests of relevance and what benchmarks exist to guide therapy and quality of care compared to nontrauma ICU patients?**

Sepsis post‐trauma operative care includes all the following common and less common sites of sepsis: Chest—pneumonia (community or ventilator/hospital acquired [VAP]) may also be seen as trauma‐associated pneumonia, catheter‐related (intravenous site, urinary tract [CAUTI], and bloodstream [CLABSI]), wound sepsis (Surgical Site Infection as defined by the centers for disease control), and less common sepsis sources such as sinusitis, tracheitis, abnormal heart valve endocarditis, meningitis or brain abscess, and middle‐ear infections [1, 2, 3, 4, 5, 6]. Unusual organisms may confound the treatment strategies in trauma patients injured in natural disasters [7]. This prolongs ICU and hospital stay and increases mortality risk [8]. It is important to have a systematic clinical approach to both screening and rational use of laboratory or radiological tests to identify these septic sources. The general principles are espoused in the latest Surviving Sepsis guidelines, although these are not without challenges in application, especially in LMIC environments, with limitations in laboratory access [9, 10]. The occult nature of the septic source may occasionally require the use of selected uncommon diagnostic options, such as PET‐scanning [11].

Associated comorbidity and poorly defined benchmarks, along with many confounding issues make setting benchmarks challenging [12, 13, 14, 15, 16, 17, 18]. The consequences of ICU care also increase the risk of certain infections [19]. Surgical technique, the use of probiotics, and the use of negative pressure wound care therapy reduce surgical site infection risk [20, 21, 22, 23, 24]. Preventive strategies include “care bundles” such as the use of special endotracheal tubes to reduce VAP rates or teams for the insertion and care of central catheters [25, 26]. Infection prevention strategies include antimicrobial stewardship, sensitivity surveillance, and rational short‐course antimicrobial therapy (usually maximally 4–5 days except for specific patient or pathology categories) [27, 28, 29, 30, 31, 32, 33, 34]. The use of quality indicators may assist in identifying at risk patients [35]. Indiscriminate use of antimicrobials is not without adverse effect, including a risk for Clostridioides infection, thus rational prescribing and different treatment strategies are important [36, 37]. It appears that in trauma patients, the use of selective digestive tract decontamination does not work [38].

Screening should include comprehensive clinical evaluation of any new onset fever in excess of 38.4°C with systemic signs of inflammation. Nonseptic causes of fever must be excluded. Blood tests include white cell count (which may lag behind other markers), platelets (an unexpected drop is related to sepsis), the sending of sputum, stool, and wound specimens for culture sent to the relevant laboratory, and the use of sepsis markers, of which procalcitonin (PCT) and beta‐d‐glucan appear the most clinically relevant, since CRP and D‐dimers are almost universally abnormal in the acute post‐trauma period. Higher PCT cutoffs have been associated with better diagnostic discrimination [39, 40, 41].

Once cultures have been sent, there is often the use of empiric antibiotic therapy; however, waiting for microbiological guidance has no worse outcome in those with probable VAP not in septic shock [30, 42, 43, 44, 45, 46]. Waiting for guidance is thus advocated in less severely ill patients. Line sepsis is best treated with removal of the offending catheter. Urinary infection is best treated with bladder washouts and catheter change along with systemic antimicrobials after exclusion of asymptomatic bacteriuria, which is far more common than actual CAUTI [47].

Treatment of surgical site infection is primarily source‐control but may include antimicrobial or topical therapy determined by whether or not there are only local signs or systemic sepsis (e.g., removal of sutures and cleaning the wound vs. the need for antimicrobials), whereas deeper sepsis will require a combination of repeat surgery and systemic therapy [48]. For intra‐abdominal sepsis, this may include relaparotomy or radiological directed drainage for source control, where “on‐demand” laparotomy is preferred over planned relook procedures.

*13. Summary and recommendations:*

Sepsis screening in the postoperative period: There is moderate quality evidence to support routine application of surgical site infection and other sepsis guidelines as for other critically ill patients with the proviso that the inflammation of trauma must be considered as a potential confounding variable for a number of the sepsis markers. Clinical correlation with results of tests is highly supported and selective culture‐guided antibiotic use recommended.

Level of evidence: moderate

Recommendation grade: strong

14. Central line insertion and Urinary catheter care and removal

**PICO: In trauma patients with invasive lines, what is the role of routine line‐change in prevention of sepsis compared to no routine change?**

In an effort to reduce ICU‐acquired device sepsis, namely, CLABSI and CAUTI, the literature supports the use of personnel education, standardized aseptic techniques for insertion, and prompt device removal when no longer needed as effective against the development of infection. There is no data to suggest routine device changes or the use of chlorhexidine or other antimicrobial washes to decrease ICU‐related device infections. A recent meta‐analysis supported the use of bundles in the insertion and maintenance of central lines, advocating for prompt removal when the device was no longer needed but not addressing routine device changes to minimize infection [48]. A bundle usually consists of full barrier precautions during the insertion of a central line, cleaning of the skin with chlorhexidine, application of appropriate hand hygiene, and prompt removal when the central line or urinary catheter is no longer needed. These strategies were also noted to be effective in the reduction of CAUTIs [47, 48, 49, 50, 51].

*14. Summary and recommendations:*

Central line insertion and Urinary catheter care and removal: There is moderate quality evidence to support bundles for the insertion of central lines and urinary catheters and to support their removal on indication rather than routine line changes.

Level of evidence: moderate

Recommendation grade: strong

15. Anesthesia

**PICO: In cases requiring an anesthesia for polytrauma and major trauma procedures, what are the optimal balanced anesthesia options for best survival in this patient group?**

The administration of anesthetic medications is frequently associated with hypotension [52]. In trauma patients, cardiovascular collapse may occur due to the vasodilation and cardiac depression caused by anesthetic agents coupled with the existing hypovolemia [53]. The time of greatest risk is during induction of an anesthesia where the effects of these agents may be combined with inadequate volume resuscitation and the loss of sympathetic tone associated with anesthesia [54]. This raises two important questions: What are the safest anesthetic agents to use? and what is the most appropriate time to induce anesthesia for airway management?

Anesthetic familiarity is a key safety measure when using induction agents. There is insufficient evidence in the anesthetic literature to stipulate specific general anesthetic induction and maintenance techniques.

Ketamine is likely to be the most cardiovascular stable anesthetic induction agent available [55]. Although hypertension is more common, the use of ketamine can be associated with hypotension on induction of anesthesia and doses should be reduced in shocked trauma patients. Ketamine has been used with safety in a wide range of clinical situations, including in the prehospital setting and in resource‐constrained environments. Ketamine can be safely used in patients with brain injury [56]. Thiopentone and propofol [57] are poor choices for induction due to the magnitude of their hypotensive effects. Midazolam has a prolonged onset and can cause prolonged hypotension due to a long duration of action [58]. Etomidate, which is also cardiovascular stable, is making a resurgence.

The use of muscle relaxants was previously controversial. It is now accepted that the use of muscle relaxants improves intubating conditions and reduces airway complications. Both suxamethonium and rocuronium are acceptable agents. The intubating doses should be increased to ensure rapid onset and dense paralysis [59].

Two maintenance techniques, which likely confer the greatest cardiovascular stability, are the use of ketamine infusions [60, 61] and the use of reduced doses of volatile anesthetics [62]. Ketamine infusions are favored in situations outside the operating theater, where there is no access to an anesthesia delivery machine. Volatile anesthetics can be titrated to depth of anesthesia monitors, thereby minimizing the dose and cardiovascular impact of these agents [63]. Intravenous anesthesia with propofol is very commonly used in elective surgery, with excellent effect profiles. In trauma patients, the pharmacokinetics can be significantly altered, especially with changes in liver blood flow, which may result in dangerously high plasma concentrations and resulting hemodynamic instability [64].

Anesthetic induction and airway management often need to occur outside the operating room to secure a threatened airway, rescue a patient in respiratory failure, or even to allow control of the process of resuscitation. The decision to administer anesthesia and manage the airway outside the operating room is difficult. The benefits of control of the airway must be weighed against multiple risks, including time lost to perform anesthesia and airway management, the risks of cardiovascular compromise, and the risk of inability to secure the airway [65]. These risks are mitigated by good preparation and the use of checklists [66].

*15. Summary and recommendations:*

Induction and maintenance of anesthesia and airway management carries a risk of cardiovascular collapse, especially in the hypovolemic patient. Fluid resuscitation should commence prior to induction of anesthesia in all hypotensive patients and those normotensive patients deemed to be significantly hypovolemic. Administration of anesthesia should be delayed until the operating theater or other point of hemorrhage control unless the patient's physiology mandates otherwise. Rational choices for administration of anesthesia are ketamine induction and maintenance, with consideration of low dose volatile maintenance of anesthesia.

Level of evidence: moderate

Recommendation grade: strong

16. Drains, nasogastric tubes, and urinary catheters

**PICO: In trauma patients with intra‐abdominal injury, what is the indication for optimal type of drain device and optimal timing of removal of such drainage devices (including nasogastric tubes and urinary catheters)?**

The ERAS concept aims to remove nasogastric tubes immediately after the conclusion of elective surgery and minimize the use of drains. Where drains are essential, early removal is advocated. For the patient with major trauma, this early removal may be impossible due to the need for monitoring in the ICU or to allow for drainage of irritant fluids in the postinjury period. This may be a cause of delay to optimal mobilization and feeding. The sentinel publication on this topic was the 2008 Schein narrative review, which advocated for selective drain use and early, but appropriate removal [67]. He also made it clear that, other than an abscess cavity, that is, noncollapsible, or for bile, urine or pancreatic juice, there is little gained by routinely draining the abdominal contents and that doing so for anastomosis “protection” is actually likely to increase the breakdown risk. Subsequently, there has been a fair amount of research on the use of, type of, and timing of removal of drains in the abdomen.

To start with urinary (UC) and nasogastric (NGT) catheters, a pilot study of enhanced recovery protocols in Cape Town showed early removal of UC and NGT was possible, with a 2.5‐day reduction in hospital LOS; however, the study only included patients not requiring ICU‐admission [68]. In similar studies from India and Brazil with a small‐cohort randomized control trial, early removal of catheters and NGT were possible with shorter hospital stays; however, they also excluded ICU‐requiring patients and there is evidence that nurse‐led ICU urine catheter removal protocols are effective in earlier removal [69, 70, 71]. For patients in ICU, the UC may be needed for either drainage (bladder repair) or for monitoring output, whereas the NGT may be retained in ventilated intubated patients to allow for early enteral feeding, often in combination with prokinetic agents and supplemental parenteral nutrition [72]. Furthermore, for patients with discontinuous bowel, the NGT may be important for decompression. These patients have a higher ileus risk as well. Comments on these two aspects in the emergency laparotomy ERAS also apply to trauma [73, 74, 75].

Drainage for biliary or pancreatic leakage has been supported and also debated for many years. In the major trauma patient, the current opinion is guided mainly by retrospective single center studies and strong personal views; however, there is some moderate‐to‐low quality guidance available [76]. Trauma pancreatectomy carries higher fistula risks compared with nontrauma pancreas resection [77, 78]. Nonoperative management of major pancreatic injury is associated with pseudocyst, so early resection and drainage are advocated (see Section 8) [79]. Late drain removal is associated with more complications, although this is usually in sicker patients [80]. For pancreas injury, an optimally placed closed suction external drain is preferred and should remain in situ until the drainage contains less than 5000 IU/mL of amylase‐containing effluent [81]. Other studies from the nontrauma pancreas surgery group suggest less than 30 mL/day, provided amylase is less than 300 IU/mL, are suggested as safe drain removal criteria [82]. For biliary leaks, these typically occur toward the end of the first week and drains should be left until after that time and less than 50 mL for 2 days drains—this is discussed in detail in Section 7 of Paper 1 (https://doi.org/10.1002/wjs.70002). Drains do portend to higher complication rates but are also used in more severely injured patients. This is certainly true for combined pancreas and duodenal injury [83, 84, 85].

*16. Summary and recommendations:*

There is low quality evidence to support optimal positioning and the removal of all drains and catheters at the earliest appropriate time guided by patient stability and clinical need. Drains are not indicated for the routine drainage of the peritoneal cavity or in the presence small or large‐bowel anastomosis.

In general, drains may be removed at less than 30 mL/day for pancreaticobiliary fistula.

Nasogastric tube: low level of evidence/weak grade of recommendation

Urinary drainage: moderate level of evidence/weak grade of recommendation

Abdominal drains: low level of evidence/weak grade of recommendation

17. Abdominal closure tray and glove change

**PICO: Does the type of hand‐scrub or the changing of gloves and instruments on closure of open surgical sites reduce the surgical site sepsis?**

The need to reduce superficial SSI may be affected by the technique of surgical closure, with the use of antibiotic impregnated sutures not showing promise in this regard. How the surgeons clean their hands has traditionally been the mainstay of SSI prevention along with surgical site preparation [86]. Five minutes of chlorhexidine soap scrub is the preferred technique avoiding the use of scrubbing brushes. Evidence from elective surgery has demonstrated that in addition to changing gloves, the use of a clean set of drapes and/or new instruments during wound closure led to reductions in wound sepsis [87, 88, 89]. Previous studies were inconclusive or used multifaceted “bundles” of care or were from the elective surgery environment [90, 91, 92]. The recent ChEETAh study demonstrated in a large cohort of patients from lower‐ and middle‐income regions that change of gloves and instruments effectively reduced SSI by a statistically significant amount in both elective and emergency surgery [93].

*17. Summary and recommendations:*

There is high quality evidence to support using chlorhexidine hand‐scrub, changing of gloves, and using a new set of instruments for definitive sheath closure in abdominal surgery, whereas extrapolation to other surgical procedures can be cautiously advocated.

Level of evidence: moderate

Recommendation grade: weak

18. Type of temporary abdominal closure device and delayed closure

**PICO: 18a In trauma patients with an open abdomen, do commercial systems versus. homemade TAC have a preferential role? 18b What is the role of mesh‐mediated traction versus. skin only in delayed closure?**

Leaving the abdomen open using a temporary abdominal closure device (TAC) may be indicated in major trauma and nontrauma surgical emergencies such as abdominal sepsis, vascular surgical emergencies, and severe acute pancreatitis. Indications include prevention and treatment of abdominal compartment syndrome, and when a second laparotomy is planned or the abdominal wall cannot be closed. There is convincing evidence that any TAC should include some form of negative pressure wound therapy, whether homemade (such as the vac‐sandwich technique) or commercial devices are used [94, 95, 96]. Focus should be on correction of physiology, infection control and nutrition, and closure of the abdominal wall as soon as possible. There is some new evidence suggesting that simple whipstitch closure as an alternative to TAC is acceptable in selected patients and increased the rates of definitive early closure in a resource challenged setting without TAC available [97].

If delayed more than 5–7 days, staged abdominal reconstruction methods include dynamic fascial traction systems combining mesh‐mediated traction with negative pressure wound therapy, yielding the highest primary fascial closure rates, and until a much later stage than the early TAC systems [98, 99]. However, awareness that dynamic traction creates IAH and prolongs the ICU stay with several relook laparotomies is important. The patient must tolerate such a physiologic challenge. The option of skin closure (with or without bridging) for later reconstruction of a hernia can be considered. Failures of primary fascial closure and enteroatmospheric fistula formation may be prevented by multidisciplinary continuity of care, planning closure as early as possible based on appropriate resuscitation with limited use of crystalloids [100]. Direct peritoneal resuscitation is currently an experimental strategy developed to improve primary fascial closure rates and reduce complications in those with an open abdomen but more convincing evidence is needed [101].

*18. Summary and recommendations:*

There is strong evidence that any TAC should include some form of negative pressure wound therapy, whether homemade or commercial.

18a. TAC should involve some form of negative pressure therapy

Level of evidence: High

Recommendation grade: Strong

18b. Abdominal reconstruction should be staged. Consider dynamic fascial traction systems with negative pressure therapy if patients tolerate it and the surgical expertize is available.

There is moderate‐to‐strong evidence supporting staged abdominal reconstruction methods, including dynamic fascial traction systems, with negative pressure wound therapy in patients who tolerate it. Mesh‐mediated traction yields the highest primary fascial closure rates and until a much later stage than the early TAC systems.

Level of evidence: moderate

Recommendation grade: weak

19a. Resuscitation/use of blood products and coagulation management

**PICO: 19a What is the most appropriate choice of resuscitation fluids for trauma patients who require fluid resuscitation from the resuscitation phase to the postoperative phase?**

The principle of intravascular resuscitation through the trauma patients' journey from resuscitation to intensive care is to restore circulating volume using fluids, which resemble, as closely as is possible, the blood which has been lost, and to replace additionally depleted factors based on either protocolized administration of blood products or directed replacement depending on the results of ongoing testing [102].

The majority of trauma systems worldwide have specific protocols for transfusion of patients following major injury, usually including aspects of fluid restriction/permissive hypotension in the presurgical phase, with restoration of volume after hemorrhage control. These systems describe the activation; administration, use of ratios of packed red blood cells, plasma, and platelets; the use of coagulation adjuncts; the management of the metabolic factors, which contribute to normal coagulation; and the endpoints of resuscitation [103]. As this is a large and rapidly evolving area of the scientific literature, these protocols require frequent updates and modifications. Adaptation to LMICs with limited blood resources also should be considered.

Retrospective analyses of civilian trauma registries and retrospective studies have shown an association between the use of whole blood and improved survival in trauma patients [104, 105]. Prospective studies are awaited before recommendations can be made regarding the use of whole blood as the primary resuscitation fluid in trauma patients. Considering blood component resuscitation, the timing of plasma administration and the ratio of plasma to red cell administration appears to be important. Early plasma administration in the prehospital environment has resulted in improved survival, in the context of long transport times [106].

The optimum ratio of red cells to plasma is 1:1 during ongoing bleeding [107]. This is consistent with the principles outlined above. Administration of reduced ratios of plasma to red cells, such as 1:2, results in a more deaths from exsanguination in the first 24 h [108]. The overall effects on mortality of these reduced ratios is less clear [109].

The use of crystalloid fluid resuscitation in trauma patients is associated with significant morbidity, especially in high doses [110]. However, profound and prolonged hypotension cause cardiovascular collapse and organ dysfunction, respectively. The role for crystalloid resuscitation is restricted to hypotensive patients, prior to the availability of blood products, to prevent cardiovascular collapse and medium‐term organ dysfunction, whereas current evidence advocates against the use of synthetic colloids for trauma patients [111, 112, 113, 114].

Following hemorrhage control and patient transfer to an intensive care environment, the clinical focus shifts to a more directed approach to blood component therapy. In this regard, the patient's hemoglobin concentration can be specifically targeted and coagulation defects addressed separately [115]. Hemoglobin transfusion thresholds in trauma have traditionally been higher than the traditional 7 g/dL in the hours to days after injury, due to the risk of ongoing bleeding or rebleeding, reoperation, and the expected reduction in hemoglobin following massive transfusion, considering trauma patients and those planned for further surgery were excluded from these studies [116, 117]. Additionally, recent studies in patients with traumatic brain injury, demonstrate that transfusion to a higher threshold of at least 9 g/dL results in improved neurological outcomes [118].

*19a. Summary and recommendations:*

Trauma patients should be resuscitated according to a locally relevant massive transfusion protocol. This protocol should aim to restore, as near as possible, whole blood to the bleeding patient. As part of these protocols, plasma components should be administered as early as possible. Ideally, these should be guided by dynamic coagulation assays, such as thromboelastography, if available. Crystalloid should be used as a last resort.

Following hemostasis, initial hemoglobin targets of greater than 9 g/dL are rational, especially where the patient has an associated brain injury, or is at risk of ongoing/rebleeding, or requires further surgery. Lower transfusion thresholds (7 g/dL) may be considered in the subsequent phases of care.

Level of evidence: moderate

Level of recommendation: strong

**19b. PICO: What is the role for supplemental medications and procoagulant blood products in the trauma patient?**

Moderate fibrinolysis may be present in as many as half of all major trauma patients and, when present, is associated with increased mortality [119]. Tranexamic acid, administered to all patients with bleeding or at risk of major bleeding, reduces mortality, especially death due to exsanguination [120]. Two significant concerns with the universal use of tranexamic acid are that fibrinolysis shutdown is more common than hyperfibrinolysis and tranexamic acid will exacerbate this, and that the improved mortality comes at the cost of more survivors with a very unfavorable neurological outcome [121].

Early fibrinogen depletion is a hallmark feature of the coagulopathy found in patients following major injury and is associated with increased mortality [122]. Early fibrinogen administration, in the form of cryoprecipitate, is feasible, but the largest trial of early empiric fibrinogen supplementation did not show a mortality benefit of routine early administration to patients with major injury [123, 124]. Fibrinogen can also be delivered by administration of factor concentrates. This method is faster than using cryoprecipitate [125]. There are concerns that protocol‐based massive transfusion protocols do not consistently address individual patients' coagulopathies [126].

In systems, which use testing‐directed transfusion protocols, point of care coagulation tests can guide the use of fibrinogen and factor concentrates. This allows for the near normalization of the patients' coagulation profile [127]. This may reduce blood product usage, but the effects on mortality are unclear [128].

*19b. Summary and recommendations:*

The use of tranexamic acid should be considered in different trauma systems depending on prehospital transfer times and access to point of care coagulation tests or dynamic coagulation assays, such as thromboelastography, if available. Cryoprecipitate is not beneficial given empirically in the early phase, whereas fibrinogen and other factor concentrates, may be administered based on tests of coagulation, as close to the point of care as possible.

Level of evidence: high

Level of recommendation: strong

**20. Ventilation in the operation room**

**PICO: What is the best management strategy for trauma patients in the operating theater—Does ARDSnet apply prior to ICU admission?**

Ventilator management strategies for trauma patients in the operating theater are aimed at preventing lung injury, optimizing oxygenation and ventilation, and reducing complications. According to a systematic review by Jaber et al. (2012), the main strategies include protective lung ventilation, individualized positive end‐expiratory pressure (PEEP), and recruitment maneuvers [129]. Protective lung ventilation consists of using low tidal volumes (6–8 mL/kg of predicted body weight), moderate PEEP (6–10 cm H_2_O), and limiting plateau pressure (< 30 cm H_2_O). This approach reduces the risk of ventilator‐induced lung injury and atelectasis, improving oxygenation and compliance. However, there is some evidence in trauma that allowing up to 10 mL/kg is better for reversal of metabolic acidosis and has no worse outcomes on the lungs [130].

A recent meta‐analysis included 17 RCTs (2250 patients). A higher driving pressure (the difference between PEEP and plateau pressure) was associated with the development of postoperative pulmonary complications (OR: 1.16, 95% CI: 1.13–1.19, and *p* < 0.0001). Increased PEEP that results in an increase in driving pressure is associated with more postoperative pulmonary complications (OR: 3.11, 95% CI: 1.39–6.96, and *p* = 0.006) [131]. The evidence for these strategies is mainly derived from RCTs and observational studies in patients undergoing (nontrauma) abdominal, thoracic, or cardiac surgery, who have similar risk factors and mechanisms of lung injury as trauma patients. However, there is a lack of specific studies in trauma patients, who may have additional challenges such as hemorrhagic shock, chest wall injury, and brain injury [132].

*20. Summary and recommendations:*

Evidence exists for optimal ventilator management of the trauma patient in the operating room. Best practices do include low‐to‐moderate tidal volume (6–8 mL/kg), moderate adjusted PEEP (5–8 cm H_2_O), and low driving pressures (< 15 cm H_2_O), although up to 10 mL/kg tidal volume may be considered acceptable in the operation room environment.

Level of evidence: high

Grade of recommendation: strong

**21. PICO: What is the best ventilator strategy for trauma patients requiring mechanical ventilation in ICU?**

Ventilator management is a critical component of care for trauma patients who require intensive care admission. The optimal settings and strategies for ventilator management may differ depending on the location of care, such as the operating room (OR) or the intensive care unit (ICU). Though not specific to trauma patients, several studies examined pulmonary complication risk in the perioperative period of major abdominal surgery.

A multicenter RCT by Futier compared protective ventilation with conventional ventilation in patients undergoing major abdominal surgery and found that protective ventilation reduced the occurrence of major pulmonary and extrapulmonary complications (RR: 0.40, 95% CI: 0.24 to 0.68, and *p* = 0.001) as well as the duration of mechanical ventilation and hospital stay (mean −2.45 days, 95% CI: −4.17 to −0.72, and *p* = 0.006) [133]. However, this approach was not confirmed by a subsequent RCT of 1236 patients undergoing major abdominal surgery who underwent either a lung protective strategy (6 mL/kg) or “conventional” approach (10 mL/kg) (OR: 0.97, 95% CI: 0.84–1.11, and *p* = 0.64) [134]. Currently, an ongoing RCT is planned to bring clarity to this important question [135].

*21. Summary and recommendations:*

Best practices are based on those patients undergoing major abdominal surgery. This includes low tidal volume (6–7 mL/kg), moderate PEEP, and low driving pressure (< 15 cm H_2_O).

Level of evidence: moderate

Grade of recommendation: strong

**22. PICO: Is there a role for ECMO in the management of trauma patients compared to no ECMO?**

Extracorporeal membrane oxygenation (ECMO) is a form of mechanical circulatory support that provides oxygenation and/or hemodynamic support to patients with severe respiratory and/or cardiac failure. ECMO has been increasingly used in various clinical scenarios, such as acute respiratory distress syndrome (ARDS), cardiogenic shock, and cardiac arrest. Historically, the role of ECMO in trauma patients was controversial due to multiple injuries (e.g., brain injury) and hemorrhage risk [136].

According to the international ECMO registry, ARDS is the most common indication for ECMO in trauma patients [137]. The average duration is 9 days for venovenous (VV) ECMO and 5 days for venoarterial (VA) ECMO. Overall survival from extracorporeal life support was reported at 70% and survival to hospital discharge was 61% in the total cohort (63% respiratory, 50% cardiac, and 25% E‐CPR). Adverse events are common (80%), with the most events being bleeding, infection, and thromboembolism. A recent meta‐analysis verified that traumatic brain injury should not be considered an absolute contraindication. Overall mortality was similarly 30% [138]. This was similar to the recommendation in the recent American Association for the Surgery of Trauma consensus statement [139]. It is recognized that access to ECMO may be limited in low resource settings and alternative ventilation options should be maximized.

*22. Summary and recommendations:*

ECMO should be considered a viable option for severely injured trauma patients that require advanced mechanical ventilatory support.

Level of evidence: moderate

Grade of recommendation: strong

**23. PICO: What is the role of noninvasive ventilation as a rescue therapy in the trauma patient versus invasive ventilation?**

Noninvasive ventilation (NIV) is a mode of respiratory support that delivers positive pressure to the airways without the need for an invasive airway. NIV has been widely used in the management of acute respiratory failure due to various causes, such as chronic obstructive pulmonary disease, cardiogenic pulmonary edema, and acute respiratory distress syndrome. The main advantages of NIV are that it avoids the complications of invasive ventilation, such as VILI, barotrauma, and VAP, and preserves the patient's ability to cough, communicate, and swallow. However, the role of NIV in the trauma patient is less clear, as there are potential benefits and risks associated with its use. A patient should always be awake enough to be able to remove the mask.

A systematic review studying NIV in trauma patients showed that in the appropriate population, attempts were feasible but only if immediate invasive endotracheal intubation were available for nonresponders [140]. A recent multicenter RCT studied NIV among patients with significant blunt chest trauma. Adults with high‐risk blunt thoracic injury were enrolled with a PaO_2_:FiO_2_ < 300 either < 48 h or > 48 h of admission. The study was stopped at 2 years due to futility with no significant difference in risk of intubation (OR: 0.72, 95% CI: 0.20–2.43, and *p* = 0.60). Similarly, there were no differences in the risk of pneumonia or ARDS [141]. A retrospective study focused on high‐flow nasal cannula (HFNC) among blunt injured thoracic patients and demonstrated safety and efficacy with more than 50% of patients recovering from respiratory failure without endotracheal intubation. Failures were associated with high thoracic injury scores such as those associated with flail chest and multiple rib fractures [142].

*23. Summary and recommendations:*

The use of noninvasive ventilation as a primary or rescue therapy in trauma patients is an option either as primary or a step‐down therapy. NIV appears to be a safe option for most patients and should not be reserved only for those with high‐risk injury, such as flail chest and a high thoracic injury score, who may fail and benefit from the NIV step‐down approach.

Level of evidence: low

Grade of recommendation: weak

**24. PICO: What is the best respiratory support for trauma patients with pulmonary contusion after initial assessment?**

Pulmonary contusion (PC) is a common and potentially life‐threatening complication of blunt chest trauma. PC is characterized by hemorrhage and edema in the lung parenchyma, leading to impaired gas exchange and respiratory failure. Respiratory support is a key component of the management of PC, but the optimal strategy remains controversial [142]. Little literature specific to traumatic pulmonary contusion exists and recommendations are based on general principles of pulmonary critical care and a 2012 guideline from the Eastern Association for the Surgery of Trauma (EAST) [143]. The current best practice is summarized here.

A judicious balanced fluid resuscitation that minimizes excessive crystalloid remains a best practice. NIV may be attempted in select patient populations, though it tends to fail among those with flail chest and high rib injury scores.

Invasive mechanical ventilation is indicated for patients with PC who have severe hypoxemia, hypercapnia, respiratory acidosis, or respiratory distress. The main goals of invasive ventilation are to maintain adequate oxygenation and ventilation, reduce the work of breathing, and prevent further lung injury. The main challenges of invasive ventilation are to avoid ventilator‐induced lung injury (VILI), barotrauma, and ventilator‐associated pneumonia (VAP). A lung‐protective strategy including low‐tidal volume (6–8 mL/kg of predicted body weight), moderate positive end‐expiratory pressure (PEEP) (8–15 cm H_2_O, [1/5 of FiO_2_]), and permissive hypercapnia (in those without concomitant TBI). Such a strategy decreases the risk of ARDS, a common complication of PC.

NIV (including high flow nasal cannula) can provide respiratory support for patients with PC who have mild‐to‐moderate hypoxemia, respiratory distress, or failure of oxygen therapy. The main advantages of NIV are that it avoids the complications of invasive ventilation, such as VILI, barotrauma, and VAP, and preserves the patient's ability to cough, communicate, and swallow.

*24. Summary and recommendations:*

Trauma patients who sustain a pulmonary contusion should have judicious use of crystalloid to prevent worsening hypoxemia. General supportive measures utilizing both NIV and lung protective strategies may be implemented, whereas invasive ventilation is the best for severe lung contusion meeting ARDSnet criteria.

Level of evidence: low

Grade of recommendation: weak

**25. PICO: What is the role for neuromuscular blockade agents in trauma patients in ICU versus nil?**

Neuromuscular blockade agents (NMBAs) are commonly used in the intensive care unit (ICU) for various indications, such as facilitating mechanical ventilation, reducing oxygen consumption, preventing shivering, and as an adjunct to ARDS treatment, while being part of the medical management of abdominal hypertension. However, the use of NMBAs in critically ill trauma patients can be associated with several risks and controversies.

The evidence for the use of NMBAs in trauma patients is limited and conflicting. One systematic review suggested that NMBAs may improve outcomes in patients with severe traumatic brain injury (TBI), by reducing intracranial pressure and cerebral metabolic rate, and preventing secondary brain injury [144]. However, other studies have shown that NMBAs may increase the risk of complications, such as pneumonia, deep vein thrombosis, and muscle weakness (especially in patients treated with steroids, or aminoglycoside antimicrobials). Moreover, the use of NMBAs may impair the neurological assessment and monitoring of patients with TBI and mask the signs of severe brain injury. Therefore, the use of NMBAs in patients with TBI should be individualized and based on careful weighing of the benefits and harms. Appropriate neurological (e.g., bispectral index) monitoring may be utilized if available to ensure adequate depth of sedation.

The evidence for the use of NMBAs in patients with other types of trauma, such as chest, abdominal, or spinal trauma, is even more scarce and inconclusive. NMBAs should be used only when indicated and after considering the alternatives, such as adequate sedation and analgesia. NMBAs should be used in the lowest effective dose and for the shortest possible duration, to minimize the adverse effects and the development of tolerance or resistance. The recent ROSE trial suggests that facilitation with NMBAs be subjected to 48 h of duration, and a recent guideline additionally advocated using bolus dosing and not continuous infusions [145, 146]. Doses should be reduced in patients with AKI and hepatic impairment. NMBAs may be used in patients with severe ARDS, as a part of a lung‐protective ventilation strategy. The oxygenation and the lung compliance of ARDS patients should be monitored closely and before and after the administration of NMBAs.

*25. Summary and recommendations:*

NMBAs could be used in the appropriate setting for trauma patients, but should be limited to bolus dosing for a maximum of 48 h duration. All patients should have BIS monitoring, if available, when paralyzed to ensure adequate depth of sedation.

Level of evidence: moderate

Grade of recommendation: weak

Aspiration Pneumonia

**26. PICO: In the trauma ICU patient who has an episode of aspiration, either during the resuscitation or later phases of care, what is the current best practice management and what is the role of bronchoscopy or antimicrobials in the management thereof?**

Aspiration of gastric contents, due to regurgitation and vomiting, the presence of nasoenteric tubes or loss of airway reflexes (resulting from low level of consciousness) is a distinct risk during the initial assessment phase, operative induction phase, and during ICU care [147, 148]. Brain trauma is a particular risk factor over and above the other risk factors in trauma ICU patients. Furthermore, patients with acute abdomen, major truncal trauma, and those who require emergency surgical intervention have a higher risk [149, 150]. Aspiration is common with an incidence between 30% and 40%, much of which is subclinical. Early antibiotic therapy for simple gastric aspiration is unnecessary, with no benefit shown and the potential for generating resistant organisms [151, 152]. Early bronchoscopy will allow for correct classification between fluid and particulate aspiration, allowing removal of particulate matter and bronchoalveolar lavage cultures can guide therapy, both for aspiration and unexpected early (non‐VAP) pneumonia [153, 154, 155, 156, 157]. Early antibiotic therapy for “sterile” gastric aspiration is not advocated, and selective antibiotics should be deescalated within 72 h if cultures are negative. It remains challenging to distinguish pneumonitis from pneumonia. When managed acutely, the morbidity and mortality are lower than other pneumonias in ICU [158]. Where antimicrobials are used, there does not seem to be an outcome difference between baseline first choice and escalated level of antibiotic therapy in this pathology [159]. Short courses of antibiotics, covering community acquired organisms but not anaerobes, may be indicated in cases with aspiration of stasis‐type enteric contents from beyond the stomach or where the aspiration occurs in the fed ICU patient [158, 160].

*26. Summary and recommendations:*

Aspiration Pneumonia

Aspiration pneumonia is distinct from pneumonia caused by infection. There is moderate level evidence to perform early bronchoscopy, lavage, and suction in these patients to allow differentiation and correct management. Antibiotics should not be routinely used until there is suspicion or evidence of infection.

Level of evidence for bronchoscopic lavage and suction: Moderate

Grade of recommendation: Strong

Level of evidence in delaying use of antibiotics unless evidence of infection: Moderate

Grade of recommendation: Strong

27. Analgesia/sedation and withdrawal

27a. *Analgesia and sedation (analgosedation) options in the trauma patient in ICU*

**27a1. PICO: In trauma patients in the ICU, what are the options for optimal analgesia and sedation to enhance recovery and early ICU mobility?**

There are multiple drugs used in ICU for induction, intubation, and continuing analgosedation: benzodiazepines, ketamine, propofol, dexmedetomidine, clonidine, and opioids, either alone or in combination [161, 162, 163]. Multimodal therapy is the standard of care with the aim to achieve sedation and comfort to one of various scoring systems, with the modern emphasis on the analgesia aspect, hence the term analgosedation, while attempting to spare opioids [164, 165]. It is recommended that sedation levels are regularly reviewed and the Ramsay score, RASS score, and Sedation‐activity score and Motor Activity Assessment score have been validated in adults and the Comfort score in children, although these scores are used inconsistently [166, 167].

Modern practice aims to reduce opioid and benzodiazepine exposure and replacing these drugs with suitable alternatives [168]. Ketamine has a renewed role along with more modern drugs, such as dexmedetomidine infusions, although the latter, such as propofol are associated with relative hypotension. Clonidine has a similar effect to that of dexmedetomidine. Titrated infusions to predefined sedation‐levels are the standard of care with sedation hold every 24 h provided criteria for weaning are met (reversal of reason for intubation and ventilation, improved level of consciousness, and no requirements for inotropes or vasopressors) [169]. Similar guidelines exist for children in ICU [170].

**27a2. PICO: Is ketamine a suitable combination with, or replacement for, propofol and opioids, or dexmedetomidine, for analgosedation in trauma patients in ICU?**

Ketamine is an old drug that has recently shown a resurgence in use given it has both a sedative and analgesic effect with lower side effects on the hemodynamic system than opioids, propofol, or benzodiazepines [171, 172, 173]. In children, this drug along with opioids is the best option for analgesia [174]. Ketamine alone is an effective opioid sparing analgosedative, either alone or in combination with either an opioid or propofol [173, 175]. Ketamine is also a good combination with dexmedetomidine for analgosedation [176].

Regarding intubation drugs for trauma ICU intubation, there is conflicting evidence, with some studies showing no overt outcome difference between ketamine, etomidate, or propofol, whereas other studies, more likely to include septic patients found etomidate to have higher mortality, but this was not found in purely trauma‐populations [177, 178, 179, 180, 181, 182]. In septic ICU populations, etomidate should be avoided, whereas ketamine is safe. Ketamine–propofol (Ketofol) combination was as safe as lower‐dose etomidate in the trauma ICU [183]. Propofol alone was associated with more adverse events in one of the largest studies in trauma, except when dose reductions were used [184, 185]. German guidelines prefer ketamine over propofol or etomidate as do the recent UK guidelines, in combination with fentanyl and with rocuronium as the paralytic agent of choice [186, 187, 188, 189].

**27a3. PICO: Is ketamine safe in polytrauma with TBI?**

Although the level of evidence for the use of ketamine sedation in TBI is based on small observational studies or small‐randomized studies with methodological concerns, it appears that ketamine is safe and does not increase intracranial pressure in patients with TBI polytrauma [190].

**27a4. PICO: What additional alternative opioid‐sparing analgesia options exist for selected patients reduced ileus in trauma patients?**

Ketamine and dexmedetomidine combined with intravenous paracetamol (acetaminophen) are commonly used to reduce opioid analgesia requirements in multimodal analgesia or analgosedation protocols in ICU. Nonsteroidal agents may be safe after 72 h postresuscitation provided there is no renal dysfunction or other risks for gastric bleeding. There is no convincing evidence of reduced fracture healing in modern studies [191, 192, 193]. The challenge is access to intravenous options for the ICU patient, whether traditional, or the more modern selective agents (coxibs) [194].

Another option that has recently come to prominence, especially in the context of ileus prevention or reduction is the use of intravenous lignocaine (lidocaine) infusions for pain relief. A recent systematic review of selected randomized controlled trials aimed to describe the pharmacokinetics, antinociceptive effects, antihyperalgesic effects, anti‐inflammatory effects, potential side effects, and to place the role of intravenous lidocaine in early postoperative pain management. Although the exact mechanism of action remains unclear, there are numerous theories. What seems clear is that the optimal dosing includes a loading dose of 1–2 mg/kg, followed by 1–2 mg/kg/h continuous infusion during early postoperative pain control and this clearly improves analgesia [195, 196, 197] In addition to the analgesia effect, infusions of lignocaine provide effective anti‐inflammatory and gastrointestinal properistaltic stimuli, thus making this an ideal addition to the multimodal analgesia options for trauma ICU patients and infusions may be continued safely for several days postoperatively [198]. Outside the abdomen, the infusions have been successfully used for control of rib‐fracture pain in the trauma population [199].

**27a5. PICO: How should opioids be weaned in TICU to prevent dependence?**

The opioid crisis in the high‐income countries has resulted in a search for alternative multimodal analgesia pathways; however, dependence is higher in those with “breakthrough” pain. A weaning protocol is recommended with the suggestion of decreasing the infusion rate by 20%–40%, followed by 10% every 12–24 h in patients at low risk of opioid withdrawal or 5%–10% reduction in dose per day in patients who have received high‐dose opioids for greater than 5 days, with patients on high‐dose fentanyl infusions more prone to withdrawal‐syndromes in ICU [200, 201]. Cross‐cover during weaning with less sedative drugs such as dexmedetomidine are suggested to reduce the risk of withdrawal syndromes, which can occur in up to 45% of those on either opioids or benzodiazepines for 5 days or longer [202].

**27a6. PICO: What is the optimal analgosedation weaning protocol for planned extubation in ICU to avoid prolonged ventilation of trauma patients?**

There is extremely limited evidence and none specific to trauma ICU to answer this question, but one study suggests that for an exclusively surgical patient population, the implementation of a combined protocol of spontaneous awakening and spontaneous breathing increased the rate of successful extubation [203, 204]. Another study suggested drug substitution and adequate monitoring are important in successfully weaning for extubation [205].

*27a. Summary and recommendations:*

Multimodal analgesia‐focused analgosedation incorporating nonopioid and nonbenzodiazepine drugs are recommended, with sedation levels monitored using a validated tool. Ketamine is a safe and effective primary analgosedation option, either with propofol or dexmedetomidine, or combined with rocuronium and fentanyl for intubation. Ketamine is a safe option, even for TBI patients and does not increase intracranial pressure. Dexmedetomidine and lignocaine infusions are newer safe adjuncts for opioid and benzodiazepine sparing in ICU, with advantages during weaning, which should be protocolized.

27a. Level of evidence: low

Grade of recommendation: weak

27b. *Withdrawal from drugs and substances in ICU trauma patients*

**PICO: In trauma patients in the ICU, which drugs and substances are associated with withdrawal syndromes and what is the best options to prevent and treat these withdrawal episodes?**

Drug and alcohol withdrawal syndromes have varied incidence in ICU populations; however, trauma patients appear to have a higher risk [206, 207]. Other drugs used in the ICU, such as opioids and benzodiazepines, also provide additional withdrawal risk [167]. Illicit recreational drugs (ecstasy and cocaine) can complicate the weaning of ventilation as well as giving false signs of cardiovascular or sepsis conditions. Chronic sedative use is also a complicating factor in drug discontinuation in the ICU [208, 209]. The signs and symptoms are well described. The treatment includes a selection of therapies including benzodiazepines or phenobarbital for alcohol withdrawal and methadone for opioid withdrawal [202, 210, 211]. Cocaine withdrawal responds well to topiramate [212]. Droperidol and haloperidol remain options for acute intervention for agitated delirium and for maintenance therapy there is a role for risperidone [213, 214]. Dexmedetomidine may be better than haloperidol for prevention of delirium and duration of symptoms [215]. Delirium is not easily preventable with the currently available sedatives and antipsychotics [216]. Protocols for management improve outcome [217].

*27b. Summary and recommendation:*

Preventing substance withdrawal is challenging with many confounding aspects and there are numerous options for treatment with clinical response to be assessed. Protocols improve outcome.

Level of evidence: moderate

Grade of evidence: strong

28. Thromboprophylaxis and holding of doses

**PICO: When should thromboprophylaxis in trauma patients commence and what type of prophylaxis is best in those without and with TBI? What is the role and timing of thromboprophylaxis in patients undergoing nonoperative management of high‐grade solid organ injuries? In trauma patients in the ICU, is there a role of screening for VTE? Is the potential benefit of withholding thromboprophylaxis prior to surgery outweighed by the risk of VTE complications?**

The authors quote and endorse the recently published trauma chapter of the European guidelines on perioperative venous thromboembolism prophylaxis [218] and only cite the recommendations below:

28a1. There is strong evidence to support early initiation of thromboprophylaxis in patients with no brain injury: Thromboprophylaxis should be initiated early (< 24 h) after severe trauma without brain injury and in the absence of active hemorrhage

Level of evidence: high

Grade of evidence: strong

28a2. For nonoperative management (NOM) of blunt solid organ injuries, VTE rates decrease consistently with early thromboprophylaxis but, based on conflicting results concerning delayed bleeding risk, some high‐risk patients might benefit from a 48 h delay.

Level of evidence: low

Grade of evidence: weak

## 3 Patients With Traumatic Brain Injury

28a3. In nonoperated patients with TBI and no progression of intracranial hemorrhage on the CT scan 24 h after the injury, we suggest early prophylaxis with LMWH within 48 h after injury.

Level of evidence: moderate

Grade of evidence: weak

28a4. In patients undergoing urgent neurosurgical interventions after TBI or in those at high risk of intracranial bleeding, we suggest delaying pharmacological prophylaxis on a case‐by‐case basis, balancing the risk of hemorrhage and the risk of VTE.

Level of evidence: moderate

Grade of evidence: strong

28a5. For trauma patients with TBI and a contraindication to pharmacological prophylaxis, we recommend intermittent compression devices.

Level of evidence: moderate

Grade of evidence: weak

28a6. We suggest adding LMWH when the risk of bleeding decreases. In patients with spinal cord injury, we suggest starting pharmacological prophylaxis within 48 h following trauma or surgery.

Level of evidence: moderate

Grade of evidence: strong

28a7. We suggest a total duration of pharmacological prophylaxis of 3–6 months after spinal cord injury with neurological deficit. We suggest combining pharmacological and IPC in patients with spinal cord injury and a motor deficit.

Level of evidence: low

Grade of evidence: weak

*28a. Summary and recommendation: Type and dose*

28b1. We recommend that LMWH be used rather than UFH as thromboprophylaxis after severe trauma.

Level of evidence: high

Grade of evidence: strong

28b2. We suggest DOAC as an alternative to LMWH in protecting against VTE after acute care (weak evidence).

Level of evidence: low

Grade of evidence: weak

28b3. We suggest that dose adjustment of LMWH is associated with reduced VTE in severe trauma patients compared to standard dosing, but there is inconclusive evidence to support one method over another (i.e., weight adjusted vs. anti‐Xa levels) and further research is required.

Level of evidence: moderate

Grade of evidence: weak

28b4. We *do not recommend* the use of thromboelastography (TEG) or rotational thromboelastometry (ROTEM) to stratify VTE risk for adjusting prophylaxis.

Level of evidence: low

Grade of recommendation: strong

28b5. In trauma patients, we recommend against the routine use of IVC filters for the primary prevention of VTE.

Level of evidence: moderate

Grade of recommendation: strong

*There is moderate evidence to suggest ultrasound (US) screening for DVT in high‐risk trauma patients*. Relevant papers vary widely in design and quality. One prospective randomized single center study enrolled high‐risk trauma patients to US screening on days 1, 3, and 7 (*n* = 995) versus no screening (*n* = 994) and found fewer PEs and more DVTs in the US group [219]. A retrospective cohort study from a single center showed an increase in PE rate over a 7‐year period, most likely associated with changes in hemostatic resuscitation, as other measures had not changed [220]. This underlines the importance of aggressive and early thromboprophylaxis and mobilization in high‐risk trauma patients. Several studies report DVT rates with US screening of 13%–14% in high‐risk trauma patients [221, 222]. Others, targeting total trauma populations find lower rates (2%) and they naturally question cost‐effectiveness of screening [223].

*There is very limited evidence on the effect of withholding TP doses prior to procedures*. Louis et al. published a prospective observational study focusing on DVT and relation with missed doses of TP [224]. The DVT rate in patients with missed doses was 23.5% versus 2.8% in the group with no missed doses. The rationale for withholding LMWH doses before surgery is being questioned and LMWH should be withheld only with neuraxial analgesia or neuro‐spinal surgery not for other procedures.

*28b. Summary and recommendation:*

28c1. We conditionally recommend routine US DVT screening in high‐risk trauma patients in ICU as per local guidelines.

Level of evidence: low

Grade of recommendation: weak

28c2. There is also moderate evidence to question the rationale for withholding LMWH doses for interventions other than neurosurgery or spinal procedures. Existing limited evidence suggests that withholding thromboprophylaxis doses before surgery increases DVT rates.

Level of evidence: low

Grade of recommendation: strong

29. Renal Replacement Therapy (RRT)

**29a. PICO: Does the timing (early vs. late) of RRT in trauma ICU patients impact on outcome?**

Acute kidney injury (AKI) is a frequent complication of trauma, affecting up to 30% of patients admitted to the ICU. AKI is associated with increased mortality, morbidity, and length of stay in the ICU. Renal replacement therapy (RRT) is a life‐saving intervention that can restore fluid and electrolyte balance, remove toxins, and support renal recovery in patients with AKI. However, the optimal indications and timing of RRT initiation for trauma patients with AKI remain controversial and depend on several factors, such as the severity of AKI, the presence of other organ failures, the risk of complications, and the availability of resources.

Indications of RRT for trauma patients with AKI are similar to those for other critically ill patients with AKI. The most common indications are volume overload, refractory acidosis, hyperkalemia, uremic complications, and intoxications. However, specific considerations for trauma patients include: (1) the potential for ongoing hemorrhage and coagulopathy, which may increase the risk of bleeding complications from RRT; (2) the need for aggressive fluid resuscitation and blood transfusion, which may exacerbate volume overload and worsen AKI; (3) the presence of rhabdomyolysis, which may cause severe hyperkalemia, acidosis, and myoglobinuria, and require high‐volume RRT; and (4) the occurrence of abdominal compartment syndrome, which may impair renal perfusion and function, and necessitate early RRT to reduce intra‐abdominal pressure.

The timing of RRT initiation for trauma patients with AKI is a matter of debate and depends on the balance between the potential benefits and harms of early versus late RRT. Several RCTs and meta‐analyses have been performed to examine the timing of RRT (early vs. late). Based on low‐to‐moderate evidence, early initiation of RRT had no beneficial effect on 30‐day mortality (12 studies, 4826 patients RR: 0.97, 95% CI: 0.87 to 1.09, *I* ^2^ = 29%, or death after 30 days (7 studies, 4534 participants: RR: 0.99, 95% CI: 0.92 to 1.07, and *I* ^2^ = 6%) [225]. These findings have been confirmed by other authors. Earlier RRT reduces the length of ICU and hospital stay but increases the risk of adverse events [226, 227, 228]. Hospital LOS −2.45 days, 95% CI: −4.75 to −0.14; *I* ^2^ = 10%; ICU LOS −1.01 days, 95% CI: −1.60 to −0.42; *I* ^2^ = 0%).

*29a. Summary and recommendations:*

Early initiation of RRT in critically ill trauma patients does not confer improved survival. However, patients who undergo early RRT will have a shorter length of ICU and hospital stay, with a risk of more adverse events. Traditional indications for RRT are best followed, but initiated sooner than later.

Level of evidence: moderate

Grade of recommendation: weak

**29b. PICO: What is the optimal type (CRRT vs. SLEDD vs. IHD) of RRT and current best practice indications for RRT in trauma ICU patients with AKI?**

The severely injured patient after major trauma is at high risk to develop renal impairment [229]. This is currently classified by the KDIGO system and relies on either low urine output or raised creatinine values to grade the severity [230]. Indications for RRT still follow accepted principles related to volume overload, refractory electrolyte abnormalities, refractory acidosis, uremic complications (gastritis, encephalopathy, and related affects), or where a dialyzable toxin is present. Since trauma patients in ICU may develop renal dysfunction from the initial trauma‐related hypoperfusion of the kidneys, from pigment‐type crush/reperfusion events, possibly from contrast media if preexisting renal dysfunction, or from postevent sepsis, there is a high‐risk in the trauma population for the requirement for RRT. Most recent evidence suggests a wait and watch approach till there are hard indications for RRT [231, 232, 233, 234, 235]. As to the mode of therapy, slow low efficiency daily dialysis (SLEDD) has potential benefits in the trauma population in those able to tolerate this in preference to continuous renal replacement therapy (CRRT) given the need to use heparin in most CRRT circuits and the challenges to doing other surgical interventions in these patients, which are often required [236]. For those patients who are hemodynamically abnormal, CRRT may be the option of choice to avoid further hemodynamic instability if they do not tolerate SLEDD; however, both of these modalities are better than traditional intermittent dialysis (IHD), which is poorly tolerated with a potential mortality benefit to SLEDD [237, 238, 239].

The one specific area of trauma‐related AKI that has unique aspects is related to crush/reperfusion syndrome with two types of syndrome, namely the anaerobic ischemia‐reperfusion syndrome and the anaerobic primary myorenal syndrome. Diagnosis is assisted with the use of lactate and venous bicarbonate levels [240, 241]. These are best managed without the use of sodium bicarbonate and avoidance of mannitol (shown to be nephrotoxic). Early RRT is recommended in this subgroup and earlier renal recovery is noted in the aerobic subgroup [231, 233, 242].

*29b. Summary and recommendations:*

There is no clear RRT therapy that is better than another, except for intermittent hemodialysis being poorly tolerated. An indication‐based approach is recommended, with liberal early initiation of RRT, earlier than traditional values. For crush/reperfusion syndrome, there may be a place for early fluid restriction if anuric and early RRT. Mannitol and bicarbonate therapy are unnecessary and do not prevent the need for RRT.

Level of evidence: high

Grade of recommendation: strong

30. Feeding/Bowel function in trauma ICU patients

In general, the existing nutritional guidelines for ICU patients, which include trauma patients, as issued by the American and European societies of enteral and parenteral nutrition (ASPEN/ESPEN) groups are comprehensive and supported [243, 244, 245, 246]. For the three specific PICO questions below, we offer some additional comments.

**30a. PICO: What is the optimal timing of feeding in the critically ill trauma patient with continuous gut?**

Nutritional support for critically injured patients is of paramount importance for short‐ and long‐term outcomes. In trauma patients, the preadmission nutritional state, type, and severity of the injury (‐ies) and surgical interventions undertaken, makes the decision of the route for feeding, dose, composition of supplementation, and the timing of initiation of nutrition support a complex decision. For the trauma patient with continuous gut, the guidelines for critical care nutrition from the various international nutrition societies are recommended as the best practice standard [243, 244, 245, 246]. This includes the patient with an open abdomen and continuous gut [247].

The advantages of starting enteral nutrition (EN) compared to parenteral nutrition (PN) early postsurgery and ICU admission have been well established. Parenteral nutrition is associated with increased inflammation and stress response, perturbations in glycemic control, and disruption in the microbiome when compared to EN. Nevertheless, early PN is recommended in cases where there is contraindication for EN or in patients who are malnourished before admission. The ESPEN critical care guidelines recommend PN initiation within 3–7 days for all patients where EN was contraindicated (e.g., GI dysfunction) and sooner (within 24–48 h) in those with documented malnutrition [248]. Safe enteral or parenteral nutrition can be commenced on low dose inotropes (equivalent to less than 13 μg/min of adrenaline) and once shock is reversed [249, 250, 251, 252, 253]. In critically ill patients feeding should start “low” and be advanced over 4–7 days [250, 253].

Gastric feeding carries low risk for the dreaded complications of aspiration and pneumonia, thus early trial of feeding should be undertaken with lower doses of infusion. Postpyloric tube placement can be considered in high risk patients with aspiration, altered mental status, dysphagia, high residual volume, nausea, vomiting, or failed gastric feeding [254]. This is shown to increase feed tolerance in this cohort [255]. To assist with gut motility, prokinetic agents are recommended in the guidelines and erythromycin or metoclopramide can be used and generally gastric residual measurement is to be avoided. For extubated patients who can eat, the chewing of gum, or consumption of coffee, along with prokinetics may have a positive effect on gastric motility [256, 257]. Prucalopride is a newer agent under investigation to assist with prokinesis [258]. For patients undergoing planned (relook) trauma surgery with intact continuous gut, who have already tolerated enteral feeds, the use of preoperative carbohydrate loading (drinks) till 2 h preanesthesia is recommended, provided gut motility is present [259].

*30a. Summary and recommendations:*

There is good quality evidence to support early enteral feeding using a gradual incremental protocol with nasoenteric feeding reserved for those who have gastroparesis or feed intolerance, with the routine use of prokinetics and adjunctive treatments recommended. Following established nutrition, society guidelines are supported for this goal. For planned surgery, clear carbohydrate liquids till 2 h preoperative are recommended to avoid prolonged preoperative starvation in those tolerating enteral feed.

Level of evidence: high

Grade of recommendation: strong

**30b. PICO: In trauma patients with discontinuous gut, what are the optimal feeding strategies and timing of feeding?**

Discontinuous gut (“clip and drop”) of bowel ends is a well‐described part of damage control surgery. Although the gut remains discontinuous and where this involves duodenum, small bowel, and proximal large bowel, we recommend early consideration for parenteral nutrition starting around day 3 provided physiological improvement had occurred and the resuscitation phase is complete.

Once gut continuity has been restored early enteral feeding within 6–8 h with a trickle‐feed approach is supported in the existing critical care nutrition literature [259, 260, 261, 262].

*30b. Summary and recommendation:*

Commence enteral nutrition (oral or tube‐feed) within 6–8 h postsurgery (trickle‐feed) once gut is continuous. Use parenteral nutrition by day 3–5 in those unable to achieve continuity of gut.

Level of evidence: moderate

Grade of recommendation: weak

**30c. PICO: To improve access to early enteral nutrition what therapies to prevent or treat postoperative nausea and vomiting or acute colonic ileus (“Ogilvie‐type”) are best in trauma patients?**

Postoperative nausea and vomiting (PONV) along with the onset of acute colonic dilatation are common reasons for delayed feeding in nonventilated trauma patients. Trauma patients fit the high‐risk group for PONV. For the management of PONV, the recommended therapies include multimodal and regional analgesia, parenteral paracetamol, dexmedetomidine, lidocaine infusion, dexamethasone early during surgery, and droperidol or ondansetron at the end of surgery. The recent guidelines are a consensus of multiple societies and we support this guideline [263]. Postoperative ileus (adynamic ileus) is common and there is a consensus evidence‐based guideline from the EAST group for the prevention and treatment of ileus [264]. For acute colonic dilatation, there is a role for escalating therapy, from prokinetics, to neostigmine, to flatus tube placement or bedside colonoscopic decompression, with surgery reserved for patients with signs suggesting bowel ischemia. Bowel dilated beyond 11 cm predicted the need for surgical intervention [265, 266].

*30c. Summary and recommendations*

There is moderate quality evidence to support the prophylactic administration of multimodal therapies to prevent PONV and ileus and to identify and treat ileus or pseudo‐obstruction in ICU.

Level of evidence: moderate

Grade of recommendation: weak

**30d. PICO: In trauma patients who undergo planned operative intervention from the ICU or the general ward does carbohydrate loading improve outcome?**

ERAS protocols advocate reduced fasting times pre‐elective surgery with the use of carbohydrate drinks to attenuate the inflammatory response and improve insulin requirements [259, 267, 268]. For the ICU patient who has to undergo planned surgery, provided they have continuous gut, there is no reason to avoid the same protocols. In fracture patients, there is weak evidence of improved outcomes from a single center small cohort randomized study [269].

*30d. Summary and recommendation:*

There is some evidence to support carbohydrate loading in the planned surgery patient with continuous gut and improving outcomes, with no adverse events.

Level of evidence: low

Grade of recommendation: weak

**30e. PICO: In patients after trauma who are intubated, does the feed need to be stopped more than 2 h prior to planned extubation?**

This is a controversial aspect of critical care with many authors recommending between 4 and 6 h of fasting prior to planned extubation; however, several more recent studies have questioned this practice [270, 271, 272].

*30e. Summary and recommendation:*

Weak evidence exists for reduced feed‐stoppage prior to extubation exists, and we recommend this decision be individualized per patient and in light of the underlying pathology and aspiration risk.

Level of evidence: low

Grade of recommendation: weak

31. Temperature Management

**31. PICO: In trauma patients what is the role of optimal temperature management in those with coagulopathy, hypothermia or post traumatic cardiac arrest?**

Regulated temperature management is established as an adjunct in postcardiac arrest care; however, optimal temperature control in the Trauma ICU patient is less well defined [273, 274, 275]. There appears to be no difference between lower and higher temperatures, with 36°C or low‐normothermia now recommended in recent updates and keeping temperatures below 37°C as the standard. For traumatic brain injury, the outcomes at 6 months in a number of studies was worse with hypothermia [276, 277, 278, 279, 280, 281].

In polytrauma patients without TBI, the recommendation of maintaining normothermia between 36.5°C and 38.3°C is based on current general ICU strategies [282]. Normothermia can be maintained with external devices or medications (paracetamol or nonsteroidal) taking the renal risk of nonsteroidal agents into account. The concern in the trauma patient with damage control or open abdomen is the risk of continued bleeding or coagulopathy, delaying return to the operating room [283]. This is worsened by hypocalcemia typically present in the critically ill patient [284]. In this cohort, rapid correction of temperature to normothermia is recommended with active warming of the room, the fluids, and the patient [285, 286].

*31. Summary and recommendations:*

The overall aim of perioperative management is to achieve and maintain normothermia. If the patient has had a prolonged cardiac arrest/loss of cerebral perfusion, targeted temperature management (TTM) may be beneficial provided the patient's circulation is stable enough and bleeding is controlled. Hyperthermia (temp > 38°C) is known to be detrimental in brain injury.

Level of evidence: moderate

Recommendation grade: strong

32. Mobilization

**PICO: In trauma patients, what are the indications and, are there contraindications, to early mobilization after trauma (except severe TBI and orthopedic restrictions) in ICU?**

Early mobilization of patients in the ICU is a strategy that aims to reduce the complications of prolonged bed rest, such as muscle weakness, delirium, and ventilator‐associated pneumonia. However, the feasibility and safety of early mobilization for patients who require mechanical ventilation and have suffered trauma is unclear. When excluding severe TBI and specific restrictions for orthopedic injuries, early mobilization of patients is seen as beneficial and very seldom contraindicated, even during the critical care phase [287, 288, 289]. Mandatory bedrest after solid organ injuries has been abandoned decades ago (see under solid organ injuries), so this is not a reason to delay mobility. For patients unable to acutely mobilize, early rehabilitation is recommended [290].

Though not specific to trauma patients, a Cochrane analysis included general ICU patients who underwent a mobilization program (4 RCTs and 690 patients). Based on low quality evidence, there was insufficient evidence to comment on the effect of early mobilization. This was due to mostly small sample size, lack of blinding, and variability in interventions and outcomes used to measure their effect size [291].

A systematic review of early mobilization among ICU trauma patients by Higgins et al. examined mortality, ventilator duration, ICU, and hospital LOS. Early mobilization was associated with decreased ventilator days (mean difference −1.18 days and 95% CI: −2.17 to −0.19). There was no difference in overall hospital mortality, ICU, or hospital LOS [292]. In one RCT, early mobilization was associated with an increase in adverse events (arrhythmias, altered blood pressure, and desaturation; (9.2% (early mobilization) versus 4.1% (usual care) and *p* = 0.005)) [293].

*32. Summary and recommendation:*

Trauma patients in the ICU who undergo a protocolized early mobilization program will have fewer days of mechanical ventilation. However, there is low quality evidence that early mobilization decreases the risk of delirium but is associated with more adverse events in ICU.

Level of evidence: moderate

Grade of recommendation: strong

## 4 Conclusion

This second part of the ERATIC trauma ERAS guidelines summarizes the current best evidence for the management of the major‐ and polytrauma patient in the ICU and postoperative care phase.

## Author Contributions

**Timothy C. Hardcastle:** conceptualization, methodology, data curation, investigation, validation, formal analysis, writing – original draft, writing – review and editing, project administration. **Christine Gaarder:** conceptualization, methodology, data curation, investigation, validation, formal analysis, project administration, writing – original draft, writing – review and editing. **Zsolt Balogh:** data curation, investigation, resources, writing – review and editing. **Scott D'amours:** writing – review and editing, investigation, data curation, formal analysis. **Kimberly A. Davis:** data curation, formal analysis, investigation, writing – review and editing. **Amit Gupta:** investigation, writing – review and editing, formal analysis. **Shahin Mohseni:** investigation, validation, formal analysis, methodology, writing – review and editing. **Paal A. Naess:** investigation, formal analysis, data curation, writing – review and editing. **Shanisa Naidoo:** investigation, formal analysis, writing – review and editing, data curation. **Tarek Razek:** investigation, data curation, formal analysis, writing – review and editing. **Simon Robertson:** investigation, formal analysis, writing – review and editing, data curation. **Hayaki Uchino:** investigation, formal analysis, data curation, writing – review and editing. **David Zonies:** investigation, formal analysis, data curation, writing – review and editing, resources. **Jade Whing:** investigation, formal analysis, data curation, resources, project administration, writing – review and editing. **Michael J. Scott:** methodology, data curation, supervision, investigation, formal analysis, writing – review and editing, resources.

## Conflicts of Interest

Timothy C. Hardcastle, Christine Gaarder, Zsolt Balogh, Scott D'amours, Kimberly A. Davis, Amit Gupta, Shahin Mohseni, Paal A. Naess, Shanisa Naidoo, Tarek Razek, Simon Robertson, Hayaki Uchino, David Zonies, and Jade Whing have no conflicts of interest. Dr Michael J. Scott, representing the ERAS group, has honoraria from and serves on advisory boards of Baxter, Edwards Lifesciences, Deltex, Trevena, and Merck. He also receives travel reimbursement from these companies and is Past President of ERAS USA.

## Data Availability

Data sharing is not applicable to this article as no new data were created or analyzed in this study.
